# Regeneration in a Degenerating Brain: Potential of Allopregnanolone as a Neuroregenerative Agent

**DOI:** 10.2174/156720507783018262

**Published:** 2007-12

**Authors:** Jun Ming Wang, Ronald W Irwin, Lifei Liu, Shuhua Chen, Roberta Diaz Brinton

**Affiliations:** Department of Pharmacology and Pharmaceutical Sciences and Program in Neuroscience, University of Southern California, School of Pharmacy, 1985 Zonal Avenue, Los Angeles, CA 90089, USA

## Abstract

Confronting the efficacy of a regenerative therapeutic is the degenerative environment that is characterized by neuronal loss, physical plague and glial scar barriers and inflammation. But perhaps more fundamental from a regenerative perspective, are changes in the biochemical milieu of steroid and peptide growth factors, cytokines and neurotransmitter systems. Data from multiple levels of analysis indicate that gonadal steroid hormones and their metabolites can promote neural health whereas their decline or absence are associated with decline in neural health and increased risk of neurodegenerative disease including Alzheimer’s. Among the steroids in decline, is allopregnanolone (APα), a neurosteroid metabolite of progesterone, which was found to be reduced in the serum [1,2] and plasma [3] and brain of aged vs. young subjects [4]. Further, Alzheimer disease (AD) victims showed an even further reduction in plasma and brain levels of APα relative to age-matched neurologically normal controls [1,4,5]. Our earlier work has shown that APα is a neurogenic agent for rodent hippocampal neural progenitors and for human neural progenitor cells derived from the cerebral cortex [6]. Our ongoing research seeks to determine the neurogenic potential of APα in the triple transgenic mouse model of Alzheimer’s disease (3xTgAD) as AD related pathology progresses from imperceptible to mild to severe. Initial analyses suggest that neurogenic potential changes with age in nontransgenic mice and that the neurogenic profile differs between non-transgenic and 3xTgAD mice. Comparative analyses indicate that APα modifies neurogenesis in both non-transgenic and 3xTgAD mice. Preliminary data suggest that APα may modify Alzheimer’s pathology progression. Together the data indicate that APα may maintain the regenerative ability of the brain and modify progression of AD related pathology. Challenges for efficacy of regenerative agents within a degenerative milieu are discussed.

## INTRODUCTION

The concept of regenerating the brain from neural stem cells is at once captivating and daunting. Regeneration during or following neurodegenerative disease, such as Alzheimer’s, requires that neural stem/progenitor cell proliferation, migration, differentiation, integration into neural circuits and ultimately function occur in a brain that has typically undergone a protracted process of degeneration. Confronting the efficacy of a regenerative therapeutic is the degenerative environment that is characterized by neuronal loss, physical plague and glial scar barriers and inflammation. But perhaps more fundamental from a regenerative perspective, are changes in the biochemical milieu of steroid and peptide growth factors, cytokines and neurotransmitter systems. A regenerative therapeutic strategy must address the challenge of regenerating neural circuits in various states of degeneration. The following concept paper describes challenges to sustaining neurogenesis during aging and Alzheimer’s, the changing milieu of regenerative agents, in particular neurosteroids, and the degenerative environment likely to be encountered by regenerative therapeutics.

## MAINTAINING REGENERATIVE POTENTIAL OF THE BRAIN

The adult brain has two stable regions of mitotic activity, the subventricular zone (SVZ) of the lateral ventricle and the dentate gyrus subgranular zone (SGZ) of the hippocampus. These two mitotic zones retain regenerative potential throughout the life span [7,8]. While the regenerative potential of the mammalian brain is sustained throughout the life span, the magnitude of the proliferative efficacy of neural progenitors declines with age [9-1]. The decline in neurogenic potential is evident as early as middle age and is one of the early changes in the aging hippocampus [9]. Early neurogenic decline is most likely due to an early decline in the concentration of neurotrophic factors, such as the steroids and peptides growth factors or a concomitant decline in receptor density or effector signaling [11-14]. 

Concomitant to the decline in neurogenesis in the aged and AD brain is the diminution in growth factors regulating neurogenesis [15-18]. The decrease in neurogenic growth factors appears to be a prime contributor to the reduced neurogenic potential of SGZ [11,12,19]. Recent studies demonstrated that the average concentration of several peptide growth factors, FGF-2, IGF-1, and VEGF, each showed a >50-60%% decline in the middle age vs. hippocampal levels in young rat hippocampi [11,12,20]. In addition, deprivation of growth hormone induced a decreased number of young healthy neurons and slower rate of neural stem/progenitor cell proliferation. Combined, these factors led to an accele-rated decay of local circuits likely because the major source of these growth factors are secreted from the stem/progenitor cells in a para- or autocrine fashion [21,22]. 

Our recent findings [6,23] and those of others [21] indicate that the neurosteroid allopregnanolone (APα, 3α-hydroxy-5α-pregnan-20-one) is a proliferative factor that could regulate the regenerative capacity of the brain. The synthesis of the neurosteroids, progesterone, and its metabolite APα in brain, first identified by Baulieu, is now well established [24-27]. A region-specific expression pattern of progesterone converting enzymes, P450scc, 5a reductase, and 3a hydroxysteroid dehydrogenase, in brain is evident in both hippocampus and cortex [26-29]. Remarkably, the enzymes 5α-reductase and 3α-hydroxysteroid dehydrogenase, required to convert progesterone to its 3a metabolites, are present and functional in pluripotential progenitors [30,31]. 

Interestingly, APα is produced within multipotential cells that also decline with age and disease. In the aged and AD brain, both the pool of neural stem cells (NSCs) and their proliferative potential are markedly diminished [2,32]. In parallel, APα content is diminished in the brains of AD patients compared with age-matched controls [5,33]. APα, with a steroidal chemical structure and low molecular weight of 318, is a reduced metabolite of progesterone. During fetal development, APα is synthesized throughout the embryonic period, is present in multipotential progenitor cells [21,30] as well as in neurons [34,35] and reaches its highest concentration in late gestation [36]. APα also can be generated *de novo* in the CNS [37,38] independent of maternal supply and of the hypothalamic-pituitary-adrenal axis.

Several strategies for maintaining regenerative capacity of the brain are reasonable viable therapeutic approaches. First is a drug based neurogenic factor replacement therapy and second is a cell based approach to replace diminished stores of neural progenitors. These strategies are discussed below. 

## CHALLENGES OF REGENERATIVE THERAPEUTICS FOR ALZHEIMER’S DISEASE

While the therapeutic potential of neural stem cells is great, so too are the challenges. AD is a diffuse degenerative disease with pathology and neuronal death occuring in multiple brain regions. Four regions within the AD brain show evidence of aberrant entry into the cell cycle predictive of neuronal loss the hippocampus, subiculum, locus co-eruleus and dorsal raphe nucleus [39]. In addition to these sites, is the late stage neuron loss of cholinergic neurons likely due to the loss of trophic survival factors retrogradely transported from the hippocampus to cholinergic neurons of the nucleus of Maynert [40,41]. Adding to the spatial complexity of neuronal loss is the phenotypic diversity of neurons targeted for demise. The spatial and phenotypic diversity of degeneration in AD predicts that a multipotent neural stem or progenitor cell population will be required for a regenerative therapeutic efficacy. 

**Fig. (1):**
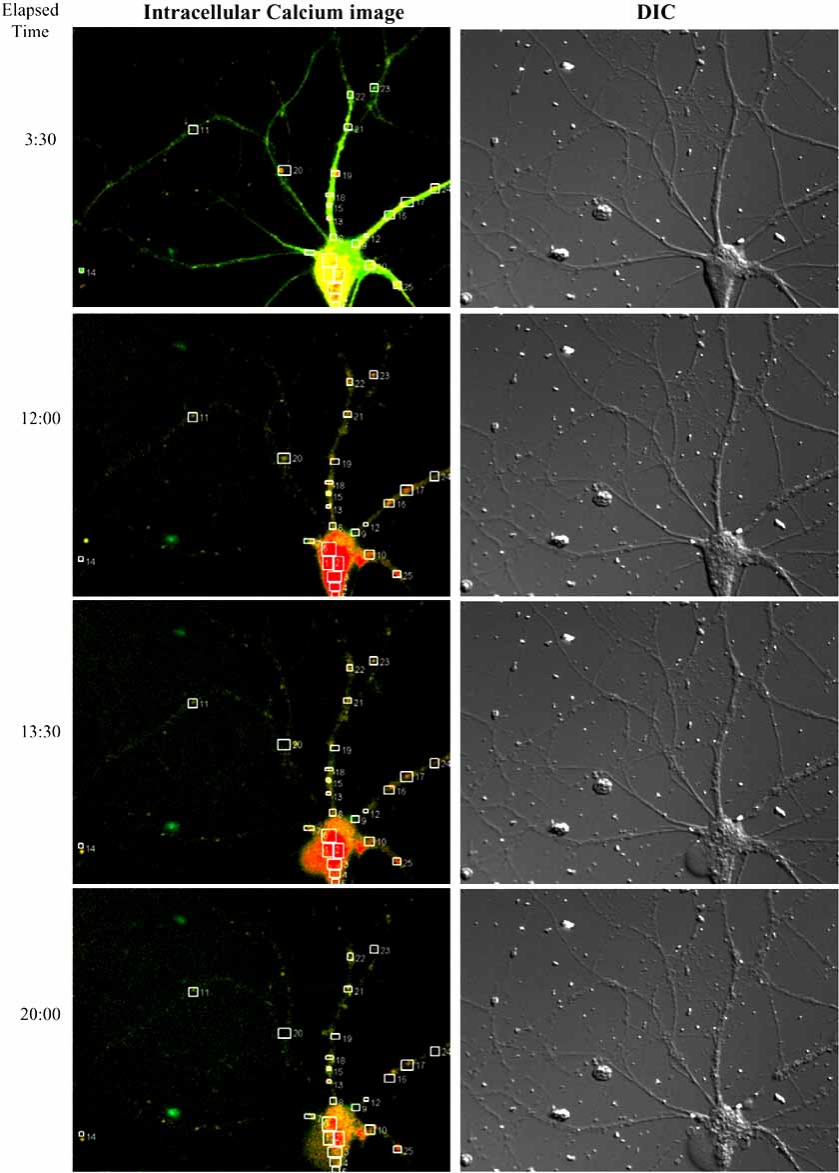
**Neurodegenerating hippocampal neuron and circuit in the presence of β amyloid and distribution of beta amyloid-induced hotspots for intracellular calcium rise.** Cultured hippocampal neurons were exposed to beta amyloid (Aβ, 4 µm) and neuron imaged over 12 hours. (DIC images in the right). Fluorescent calcium imaging indicates an intense rise in intracellular calcium that the cell body that is sustained over the course of 90 minutes and proceeds the loss of plasma membrane integrity as manifested by cell body ballooning apparent at bottom left of cell body. In DIC images in the right column, degeneration is morphologically apparent 13:30 with the ballooning of the cell body. At 20:00 hr intracellular calcium has declined and the neurodegeneration of both the cell body and neurites is apparent. These observations suggest that the prolonged intracellular rise in calcium induced by Aβ is an event coupled to degeneration and is likely to be the cause of degeneration in Aβ-exposed neurons.

In addition to the diversity of local and phenotype of degenerating systems is the topographical landscape of the degenerating terrain. The degenerative milieu of AD is characterized by an increased number of neuritic plaques and neurofibrillary tangles in the cerebral cortex [42]. The former (neuritic plaques) are composed of tortuous neuritic processes surrounding a central amyloid (Aβ) core. The later is characterized by the abnormal hyperphosphorylation and accumulation of tau protein in neurons and, less commonly, in astrocytes. The neurofibrillary tangles, formed by abnormal hyperphosphorylated tau, are frequently seen AD brain and accompanied by neuronal loss and gliosis [43]. Although the presence of increased numbers of neuritic plaques and neurofibrillary tangles in neocortex is necessary for a diagnosis of AD, they are also found in the hippocampus, which induce the dysfunction and loss of hippocampal neurons [44,45]. Inflammation also plays an important role in pathogenesis of neurodegenerative disorders including AD [46, 47]. In AD brains, compacted Aβ plaques are often associated with activated astrocytes and microglia and a variety of cytokines and other inflammatory proteins secreted by activated astrocytes or microglia, including Clq, C3, C9, C3d, and C4d, which are found in brains from human AD patients and mouse models of AD [48-50]. Thus, the regenerative stem cell population must survive and traverse a landscape riddled with degenerative debris and replete with a biochemical cauldron of inflammatory, cellular stress and defense molecular signaling (see Fig. **1**).

Last but not least, is the disturbing finding from multiple laboratories indicating that ectopic entry into the cell cycle is an early marker of AD and predicts the cells that will meet an untimely death. Cell cycle gene expression in neural progenitor cells is an obligatory requirement for neurogenesis and ultimately regeneration. However, neurons within the cortex and hippocampus can aberrantly reenter the cell cycle [51-53] and ectopically express cell cycle proteins [54,55]. Most disturbing of all, Herrup and colleagues found that cell cycle events precede neuronal death in the cortex and CA3 regions at all stages of AD, from MCI to late stage AD and within AD mouse models [52,53]. Expression of the ectopic cell cycle proteins ultimately predicts the demise of these neurons [52,53]. Further support for the aberrant entry into the cell cycle and cell death in AD are findings indicating that mutants of APP known to cause familial AD also lead to apoptosis and DNA synthesis [56-59]. These findings are especially challenging for therapeutics targeting regenerative potential of endogenous neural stem / progenitor populations as an unintended side effect may be to promote ectopic entry of neurons into the cell cycle and thereby exacerbate neuron demise.

## APα AS A REGENERATIVE FACTOR TO PROMOTE FUNCTIONAL NEUROGENESIS AND DIMINISH ALZHEIMER’S PATHOLOGY

Recently, we demonstrated that APα promoted *in vitro *proliferation of human and rat neural progenitors and mouse hippocampal neurogenesis *in vivo *in a dose dependent and steroid specific manner [6]. The proliferative effect of APα we observed in rat hippocampal neural progenitor cells and human cerebral cortical neural progenitor cells *in vitro* [6], was also observed in rodent cerebellar granule cells which also undergo proliferation during development [60]. APα induced neural progenitor proliferation ranged from 20-30 % in the rodent neural progenitor cells to 37-49% in the human neural stem cells [6]. The efficacy of APα as a neurogenic factor was comparable to that induced by bFGF + heparin [6]. Our analyses demonstrating that APα increased BrdU incorporation are consistent with gene array and real time RT-PCR data. APα increased expression of genes that promote transition through the cell cycle and proliferation, such as cyclins and CDKs including CDC2, cyclin B and PCNA. Correspondingly, APα down regulated the expression of genes involved in inhibition and degradation of CDKs and cyclins, such as CDK4 and CDK6 inhibitor P16, P18, cullin 3 and ubiquitin-activating enzyme E1(Ube1x), enzymes that are required for ubiquitination of mitotic cyclins and promote exit from the cell cycle. Consistent with APα-induced cell cycle gene expression and BrdU incorporation, APα increased total cell number. APα-induced neurogenesis was a dose dependent process with concentrations within the low to mid 10^9-7 ^range promoting proliferation while concentrations in excess of 10^6^ significantly inhibiting neurogenesis. In immature rat cerebellar granular cells APα induced ~ 20% increase in thymidine incorporation and a 20-30% increase of PSA-NCAM positive progenitor proliferation derived from rat brain [21,60]. Together, these data indicate that APα can promote neurogenesis of neural progenitor cells derived from multiple sites and diverse phenotypes. 

To determine whether our *in vitro* findings were recapitulated *in vivo*, we used the triple transgenic Alzheimer’s disease (3xTgAD) mouse, developed by Dr. Frank LaFerla, as both a model of AD pathology and as an animal model for assessing therapeutic efficacy. The 3xTgAD mouse possesses mutations in three genes linked to AD and frontotemporal dementia [44,45,61-63]. This mouse model develops age and pathology dependent synaptic dysfunction, Aß plaque and neurofibrillary tangle pathologies as well as the accompanying astrocytic response (GFAP increased around plaque) [61,64-66]. There are several advantages to this model. First, the tight APP and tau linkage paired with the 'knock in' PS1 approach yielded homozygous mice. Second, and more importantly, the 3xTg-AD mouse exhibits an age-related neuropathological phenotype that includes both intracellular an extracellular Aβ deposition and hyper-phos-phorylated tau pathologies that develop in an age-dependent fashion with a regional pattern similar to AD. Specifically, Aβ accumulates first intracellularly and then extracellularly in cortical regions and in hippocampus while tau hyper-phosphorylation develops after Aβ accumulation (between 12-15 months) beginning in limbic structures and progressing to cortical regions [61]. Confirming and characterizing the *in vivo* neurogenenic effects of APα on this animal model will create the foundation upon which we will investigate the relationship between the APα-induced neurogenesis and associated behavior as well as regulation of AD pathology. 

**Fig. (2):**
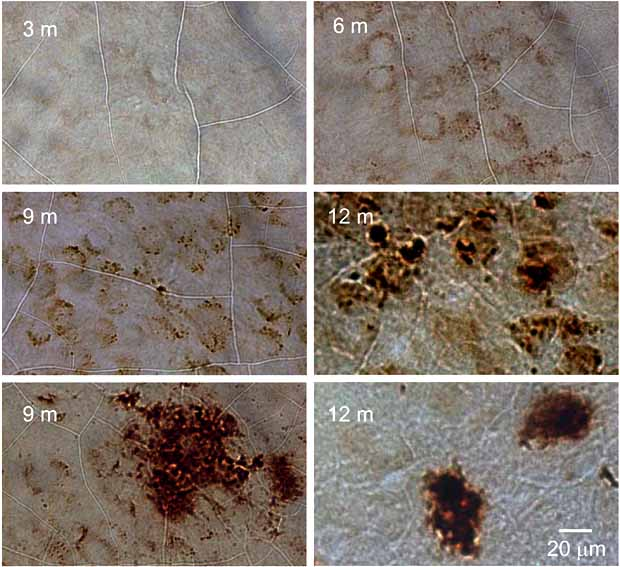
**Age dependent AD pathology development in 3xTgAD mouse hippocampal CA1.** 3xTgAD mouse were perfused and sampled at different ages as indicated. The mouse brain sections were immunostained with anti-Amyloid β antibody and observed with peroxidase-DAB. The results indicated an age-dependent development of Aβ pathology in CA1 of the mouse hippocampus. At 3 months, cellular Aβ IR is barely visible. At 6, 9 and 12 months, intracellular Aβ IR intensity increased with age. Extraneuronal Aβ IR was observed rarely in 9 month old 3xTgAD hippocampi but was consistently present in hippocampus of 12- month-old 3xTgAD mice.

**Fig. (3):**
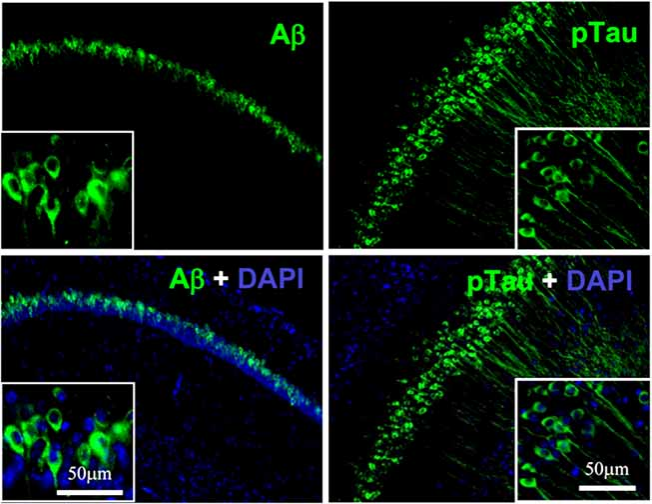
**Beta amyloid and ptau expression in six month old 3xTgAD male mouse CA1 region of hippocampus.** 3xTgAD mice were perfused with PBS and fixed in 4% PFA for 16 hours. Mouse brain hemispheres were embedded and were sectioned to 40 µm slices. Aβ IR was primarily localized neuronal cell bodies. Phosphotau was labeled with a phospho-tau specific antibody. Insert = high magnification.

Our preliminary *in vivo* analyses suggest that APα can increase BrdU incorporation in 3xTgAD mouse SGZ [23,67] as well as the SVZ [23,67]. Our initial analyses were conducted in 3 month old 3xTgAD mice prior to the appearance of pathology associated with AD in these mouse (see Figs. **2** and **3**). At later ages, APα also significantly reduced the level of Aβ in the CA1 region in 6-month-old-male mice after a treatment for 3 months. Phospho-tau in the CA1 region in 9-month-old males was reduced following acute treatment APα treatment. 

Mechanistically, APα is a potent and stereoisomer specific allosteric modulator of the GABA chloride channel complex and in neural progenitor cells increases conductance through the channel which can be protective against seizure activity [6]. In neural progenitor cells, the high intracellular chloride content leads to an *efflux* of chloride through the GBRC, depolarization of the membrane and opening of L-type voltage dependent Ca^++^ channels [6]. APα transiently increased the intracellular calcium concentration in both rat and human neural progenitor cells. The APα-induced rise in intracellular calcium was blocked by GABA_A_R inhibitors, bicuculline and picrotoxin as well as the L-type calcium channel antagonist, nifedipine. In parallel to the antagonism of intracellular calcium concentration, nifedipine also blocked APα-induced cell proliferation [6,68]. 

Our preliminary findings suggest that APα also reduces AD pathology burden. This finding is consistent with the findings of Mellon and colleagues who reported that APα induced significantly delayed progression of disease in a mouse model of Niemann-Pick C disease [69-71]. Niemann-Pick C disease is an irreversible inherited neurodegenerative disorder involving a deficient intracellular cholesterol and/or ganglioside traffics [72,73]. Mutations in either the Niemann-Pick C1 or Niemann-Pick C2 gene encodes dysfunc-tional proteins which lead to abnormal binding and movement of cholesterol and lipids within cells and accumulation of unesterified cholesterol within lysosomes and the Golgi apparatus [74-76]. In young animals, either single or multiple injections of APα protected cerebellar Purkinje cells from degeneration and increased animal life span. Less improvement was observed at older ages of Niemann-Pick C1^–/–^ mouse that had disrupted neurosteroidogenesis [70]. Langmade, Gale, and colleagues reported that the APα-induced a delay in progression of pathology and enhanced survival of Niemann Pick C mice was through a pregnane X receptor receptor-mediated mechanism. The pregnane X receptor (PXR) is a nuclear receptor that binds to various ligands, regulating the breakdown of drugs in the human body. PXR is activated by a large number of endogenous and exogenous chemicals including steroids, antibiotics, antimycotics, bile acids, and herbal antidepressants. One of the primary functions of PXR activation is the induction of CYP3A4, an important enzyme responsible for the metabolism of many drugs. Mechanistically, APα activation of the PXR receptor, leading to an increase in the cytochrome P450 enzyme CYP3A13, suggests a novel mechanism of APα neuroprotection that brings the benefits of "liver" detox to the brain. Therapeutically, activating “liver-type” detoxification in brain may be a broad-spectrum stra-tegy for promoting neurological health and defense against neurodegenerative insults.

## THERAPEUTIC POTENTIAL OF APα HAS A RE-GENERATIVE FACTOR

Unlike large molecular weight growth factors, such as FGF and neurotrophins, which do not readily pass the blood brain barrier and induce untoward side effects in humans [77], APα with a steroidal chemical structure, 3a-hydroxy-5α-pregnan-20-one, and low molecular weight of 318.49, easily penetrates the blood brain barrier. Our discovery that APα is a proliferative agent for neural progenitor cells *in vitro* and *in vivo* suggests that APα could act to promote proliferation in the AD brain. Further, the very preliminary finding that APα could delay or diminish AD pathology burden suggests that APα could be a multifaceted regenerative therapeutic to both promote the mechanisms of cellular regeneration while diminishing the degenerative barriers to regeneration. 

A neurodegenerative disease that may serve as an initial proof of concept for neural stem cell mediated regeneration is Parkinson’s disease. Compared to AD, neurodegeneration in Parkinson's disease is more tractable, as degeneration of a specific type of neuron in a specific locale, dopaminergicneurons in the substantia nigra, occurs. The most successful clinical example for using progenitor cell or embryonic tissue to combat neurodegenerative disease is the implantation of embryonic fetal mesencephalic tissue into a cavity of Parkinson's disease patient’s caudate nucleus. Results of these trials indicate different magnitudes of benefit that remained apparent at 5 -10 years following cell implants [78-82].

Our therapeutic approach would be to forego cell implants for promoting endogenous proliferation of neural progenitor cells within the brain, which, although in low abundance, could be induced to proliferate. Moreover, we advocate a small molecule approach, rather than large molecular weight peptide growth factors which are unlikely to cross the blood brain barrier. Thus far, APα appears to be a promising regenerative therapeutic candidate for promoting cellular regeneration in a neurodegenerative disease that requires regeneration in multiple sites and of multiple neural phenotypes and for diminishing the degenerative pathology burden. Our future work will pursue these issues.
